# Large area growth of MoTe_2_ films as high performance counter electrodes for dye-sensitized solar cells

**DOI:** 10.1038/s41598-017-18067-6

**Published:** 2018-01-08

**Authors:** Sajjad Hussain, Supriya A. Patil, Dhanasekaran Vikraman, Naveed Mengal, Hailiang Liu, Wooseok Song, Ki-Seok An, Sung Hoon Jeong, Hak-Sung Kim, Jongwan Jung

**Affiliations:** 1 Graphene Research Institute, Sejong University, Seoul, 143-747 Republic of Korea; 2 Institute of Nano and Advanced Materials Engineering, Sejong University, Seoul, 143-747 Republic of Korea; 3 Department of Mechanical Engineering, Hanyang University, Haengdang-dong, Seongdong-gu, 133-791 Seoul, Republic of Korea; 4 Institute of Nano Science and Technology, Hanyang University, Seoul, 133-79 Republic of Korea; 5 Division of Electronics and Electrical Engineering, Dongguk University-Seoul, Seoul, 04620 Republic of Korea; 6 Department of Organic and Nano Engineering, Hanyang University, Seoul, 133-791 Republic of Korea; 7 Thin Film Materials Research Group, Korea Research Institute of Chemical Technology, Daejon, 305-600 Korea

## Abstract

A cost effective and efficient alternative counter electrode (CE) to replace commercially existing platinum (Pt)-based CEs for dye-sensitized solar cells (DSSCs) is necessary to make DSSCs competitive. Herein, we report the large-area growth of molybdenum telluride (MoTe_2_) thin films by sputtering-chemical vapor deposition (CVD) on conductive glass substrates for Pt-free CEs of DSSCs. Cyclic voltammetry (CV), Tafel curve analysis, and electrochemical impedance spectroscopy (EIS) results showed that the as-synthesized MoTe_2_ exhibited good electrocatalytic properties and a low charge transfer resistance at the electrolyte-electrode interface. The optimized MoTe_2_ CE revealed a high power conversion efficiency of 7.25% under a simulated solar illumination of 100 mW cm^−2^ (AM 1.5), which was comparable to the 8.15% observed for a DSSC with a Pt CE. The low cost and good electrocatalytic properties of MoTe_2_ thin films make them as an alternative CE for DSSCs.

## Introduction

Dye-sensitized solar cells (DSSCs) are gaining considerable interest for next-generation photovoltaic devices due to their acceptable energy conversion efficiency, low cost, environmental friendliness, and easy fabrication processes^1,2^. Typically, DSSCs have a sandwich structure with a photoanode (a semiconductor film on an FTO substrate sensitized by dye molecules), an electrolyte containing the iodide/triiodide (I^−^/I_3_^−^) redox couple, and a counter electrode (CE) catalyzing the reduction of I_3_^−^ to I^−^. Platinum (Pt) is an excellent catalyst for the reduction of I_3_^−^ to I^−^ due to its superior conductivity, electrocatalytic activity, and stability^3,4^. However, Pt is a noble metal and it is scarce and expensive. Therefore, new materials have been explored to develop cost-effective Pt-free CEs for DSSCs. To date, numerous attempts have been made to find alternative CEs, including transition metal dichalcogenides (TMDC), carbon materials, conducting polymers^5,6^, nitrides^7,8^, and carbides^9,10^. In particulary, interests in 2D materilas such as TMDC materials including selenides and sulphides are high because of their good electrocatalytic activity and stability^11–14^. Previously, our group demonstrated that molybdenum disulfide (MoS_2_) and tungsten disulfide (WS_2_) are good CE materials for DSSCs. They exhibited photovoltaic conversion efficiencies (PCEs) of 6.0% and 6.3%, respectively^15,16^. However, the efficiency is still not satisfactory, and efforts to improve the efficiency and discover a new TMDC materials are ongoing. Recently, tellurides such as WTe_2_ and MoTe_2_ in the family of TMDC materials are gaining interests in electronic and optoelectronic devices^17–19^. Like other TMDC materials, the band gap of MoTe_2_ also depends on the number of layers. MoTe_2_ has an lowest indirect band gap of ~1.0 eV, and single-layer MoTe_2_ is a direct gap material with an optical band gap of 1.1 eV^20^, close to that of Si (1.1 eV)^21^. MoTe_2_ has a low band gap in the family of TMDC materials. MoTe_2_ crystal is highly stable in semiconducting (2 H) and metallic (1 T′) phase in nature^22,23^. The hydrogen evolution reaction catalytic activity of MoTe_2_ was reported^24^. In this work, we report the catalytic activities of MoTe_2_ as counter electrode in DSSCs.

Herein, we have grown MoTe_2_ thin films *via* sputtering combined with a post-deposition annealing process on conductive glass substrates with different thickness. This work is a continuation of our research focusing on TMDC material search and growth for DSSC applications. MoTe_2_ films used as CEs in DSSC showed good electrical conductivity and electrocatalytic activity, and a DSSC employing a MoTe_2_ CE synthesized under optimized conditions had a 7.25% PCE, which is comparable to the value of 8.15% obtained for the Pt CE under the same conditions. To the best of our knowledge, this is the highest PCE for (I^−^/I_3_^−^) redox couple-based DSSCs employing MoTe_2_ CE under a simulated solar illumination of 100 mW∙cm^−2^ (AM 1.5).

## Results and Discussion

**Figure 1:**
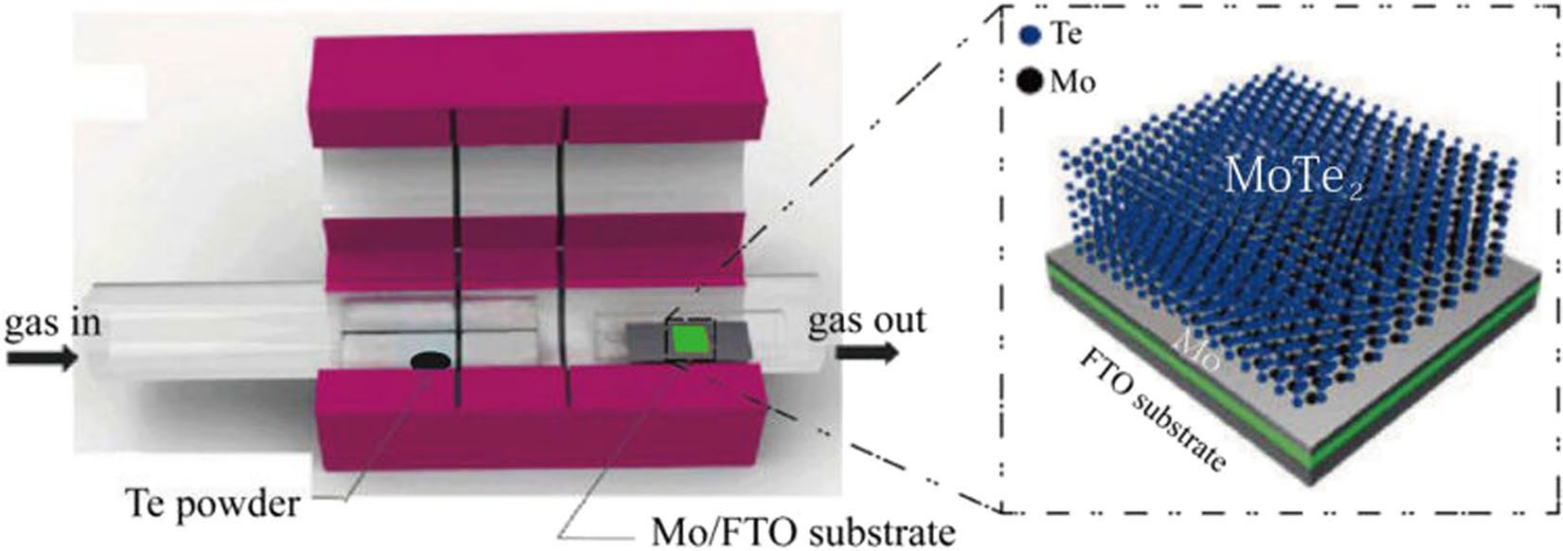
Schematic illustration of tellurization for the preparation of MoTe_2_ from Mo/FTO substrate using a two-zone chemical vapour deposition chamber.

In this study, we fabricated large-area and high-quality MoTe_2_ directly onto FTO substrate by sputtering-CVD growth, as depicted in Fig. 1. Our synthesis method consists of two steps. Initially, Mo films were deposited onto FTO substrates using magnetron sputtering, and the film was annealed at 500 °C in a tellurium environment in a CVD chamber. Three samples were sputtered at three different times (20, 30, and 40 min) and subsequently tellurized, and referred to S1 (~185 nm), S2 (~335 nm), and S3 (~668 nm), respectively.

**Figure 2:**
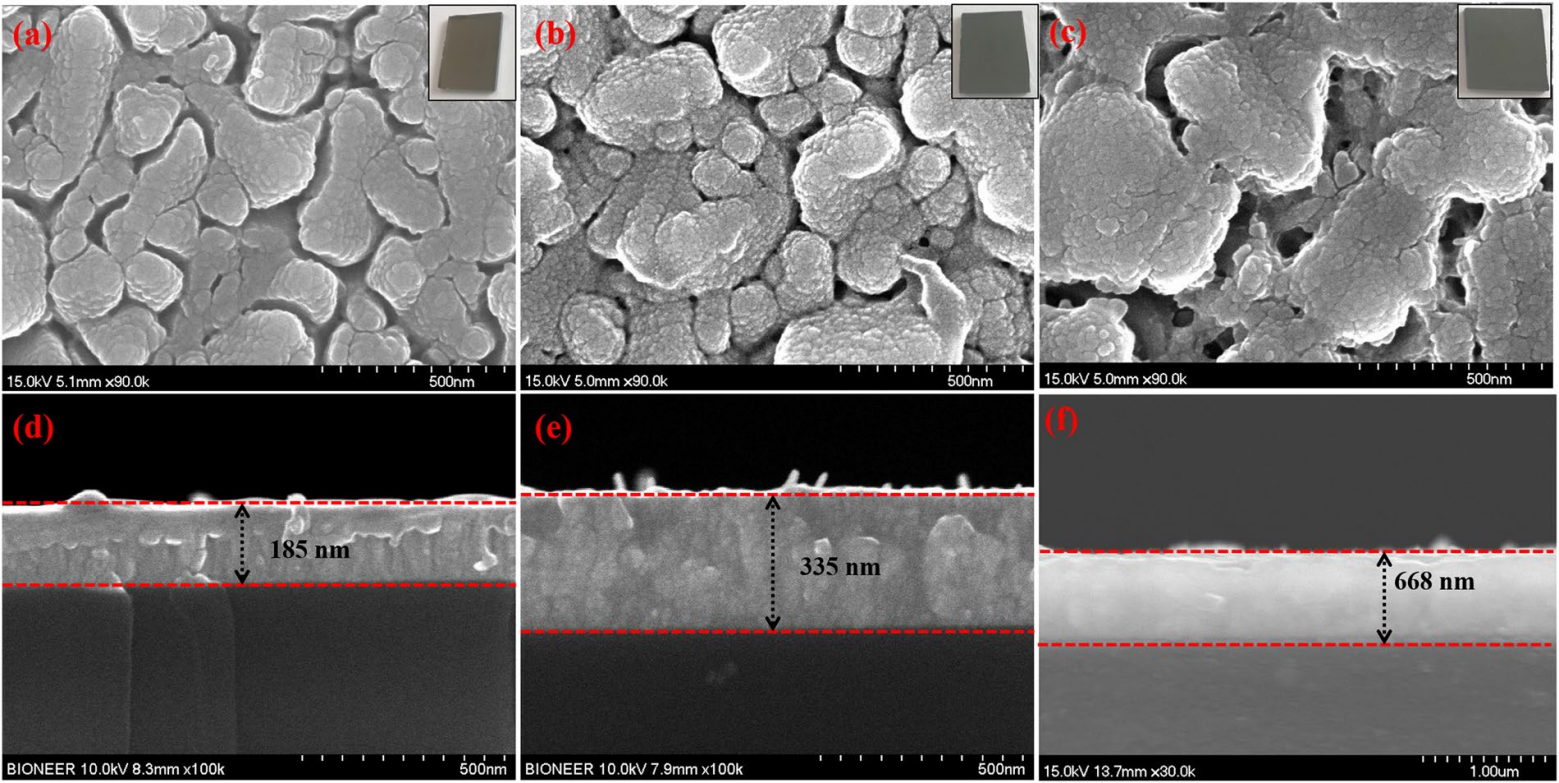
(**a–c**) Top down FE-SEM images of S1, S2 and S3 (Inset shows the corresponding image of MoTe_2_ sample) and (**d–f**) cross-sectional images of S1, S2 and S3. The observed thickness were ~185 nm, ~335 nm and ~ 668 nm for S1, S2 and S3, respectively.

Field emission scanning electron microscopy (FE-SEM) analysis was performed to reveal the surface morphology of the MoTe_2_/FTO structure. Figure 2(a–c) provide FE-SEM images of samples S1, S2 and S3, respectively. Samples exposed to the longest tellurization (40 min) exhibits the biggest grains in Fig. 2(c). Cross-sectional SEM images show that the thicknesses of the S1, S2 and S3 are ~185, ~355 and ~688 nm, respectively (Fig. 2d–f). The low magnification FE-SEM image with EDS spectrum for the sample S2 is provided in supporting information (Figure S1a,b). The cross-sectional view with their EDS profile is provided to confirm the presence of Mo and Te in the MoTe_2_ film (Figure S1c,d).

**Figure 3:**
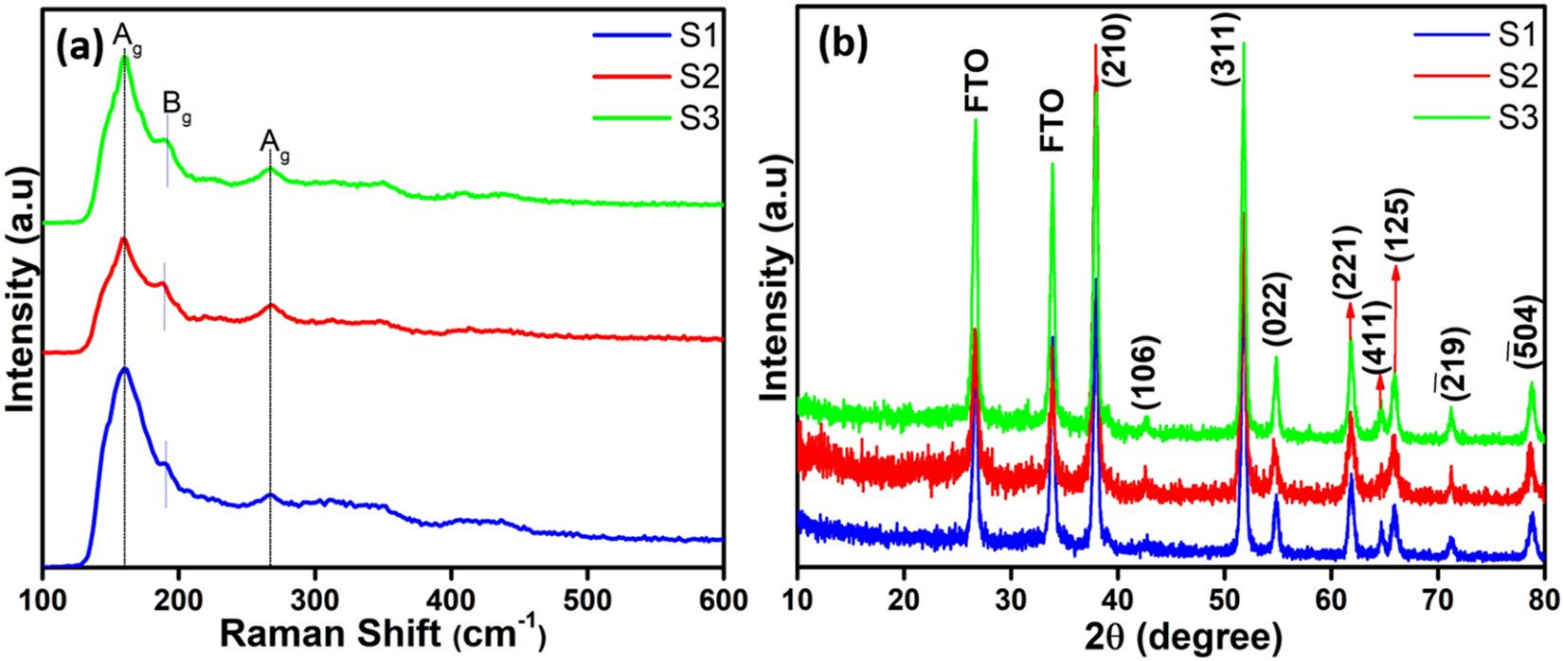
(**a,b**) Raman spectra and XRD patterns of MoTe_2_ samples.

The structures of the MoTe_2_ films were characterized by Raman spectroscopy using a 514 nm excitation laser. Figure 3(a) shows prominent peaks at ~161, and ~267 cm^−1^, which correspond to the A_g_ mode. A shoulder peak was observed at ~189 cm^−1^, and this was ascribed to the B_g_ mode, for MoTe_2_ in the 1 T′ phase. The spectrum agrees well with the previously reported results^22,25^. XRD measurements were performed to further evaluate the identity and structure of the film, as shown in Fig. 3b. The XRD patterns show that the synthesized MoTe_2_ films were polycrystalline in nature with a monoclinic structure. The diffraction peaks were at 38.0°, 42.7°, 51.7°, 54.7°, 61.7°, 64.7°, 65.9°, 71.2°, and 78.8°, which correspond to (210), (106), (311), (022), (221), (411), (125), $(\bar{2}19)$, and $(\bar{5}04)$ lattice planes of MoTe_2_, respectively (JCPDS No. 71–2157). No impurities or other reflections from deleterious crystalline phases were observed, which suggests that well oriented MoTe_2_ films were deposited.

**Figure 4:**
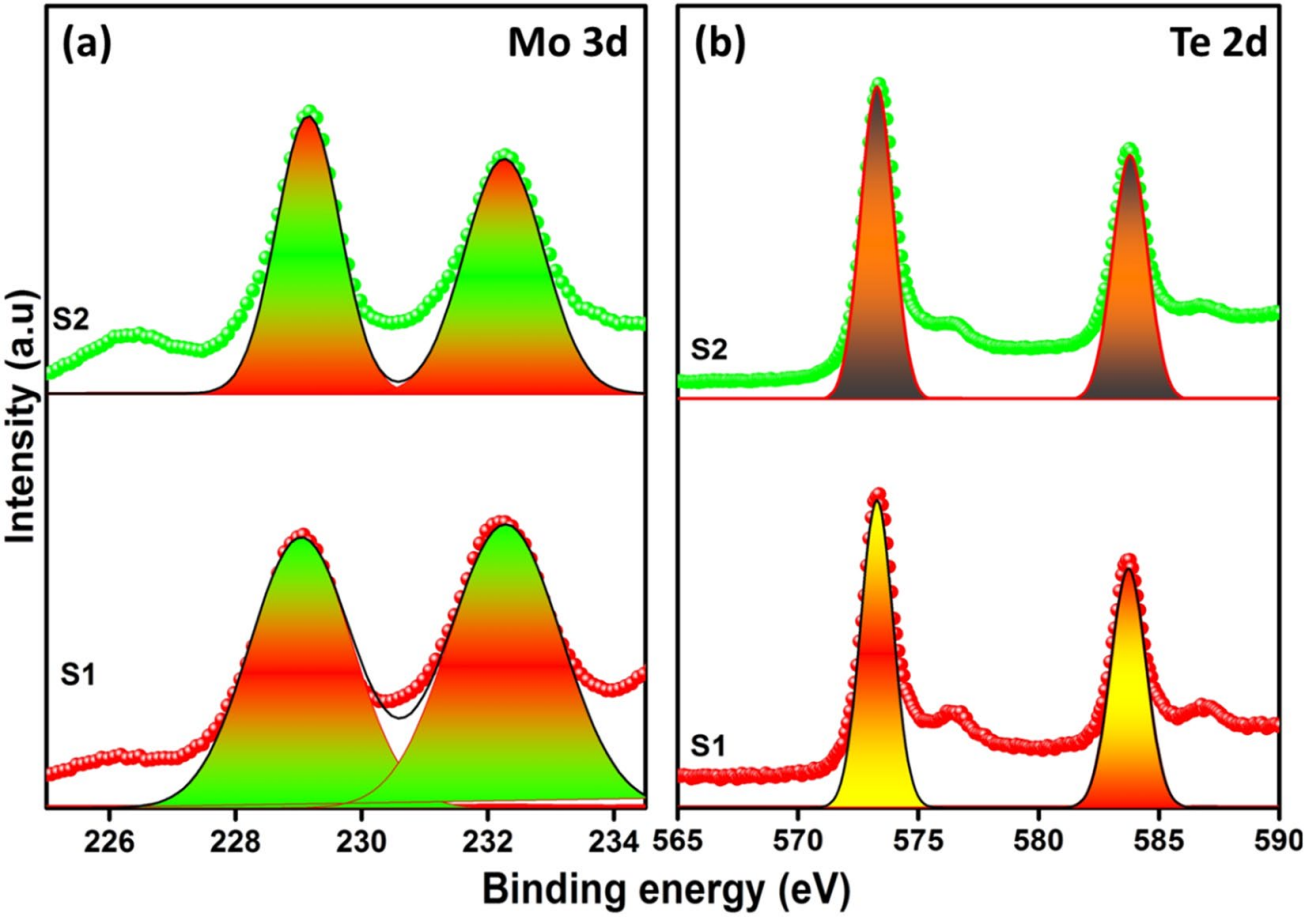
**(a**,**b)** XPS spectra of MoTe_2_ samples (**a**) Mo atoms and (**b**) Te atoms of S1 and S2, respectively.

X-ray photoemission spectroscopy (XPS) was used to verify the surface chemical compositions and valence states of 1 T′-MoTe_2_. The survey spectrum indicates the coexistence of Mo and Te elements in the MoTe_2_ films (Figure S2). High-resolution spectra of each element are also given in Fig. 4a,b. As shown in Fig. 4a, the Mo 3d spectrum exhibits two main peaks at 229.2 and 232.2 eV, corresponding to the doublet of Mo 3d_5/2_ and Mo 3d_3/2_. For Te 2d specturm, peaks were observed at 573.1 and 583.6 eV, as shown in Fig. 4b. These can be assigned to the spin–orbit couple of Te 2d_5/2_ and Te 2d_3/2_, respectively^18^. The stoichiometry of Mo and Te elements in our synthesized MoTe_2_ film is confirmed by EDS spectrum (Fig.  S1b). Hall measurements were performed on MoTe_2_/glass at room temperature (RT) with an active area of (1 × 1) cm^2^ (Figure S3). MoTe_2_ CE revealed p-type behavior similar to that reported in the literature^26^. The conductivity = 3.3 × 10^−1^ Ω^−1^cm^−1^, and charge mobility = 95 cm^2^ V^−1^ s^−1^ were extracted from the device.

**Figure 5:**
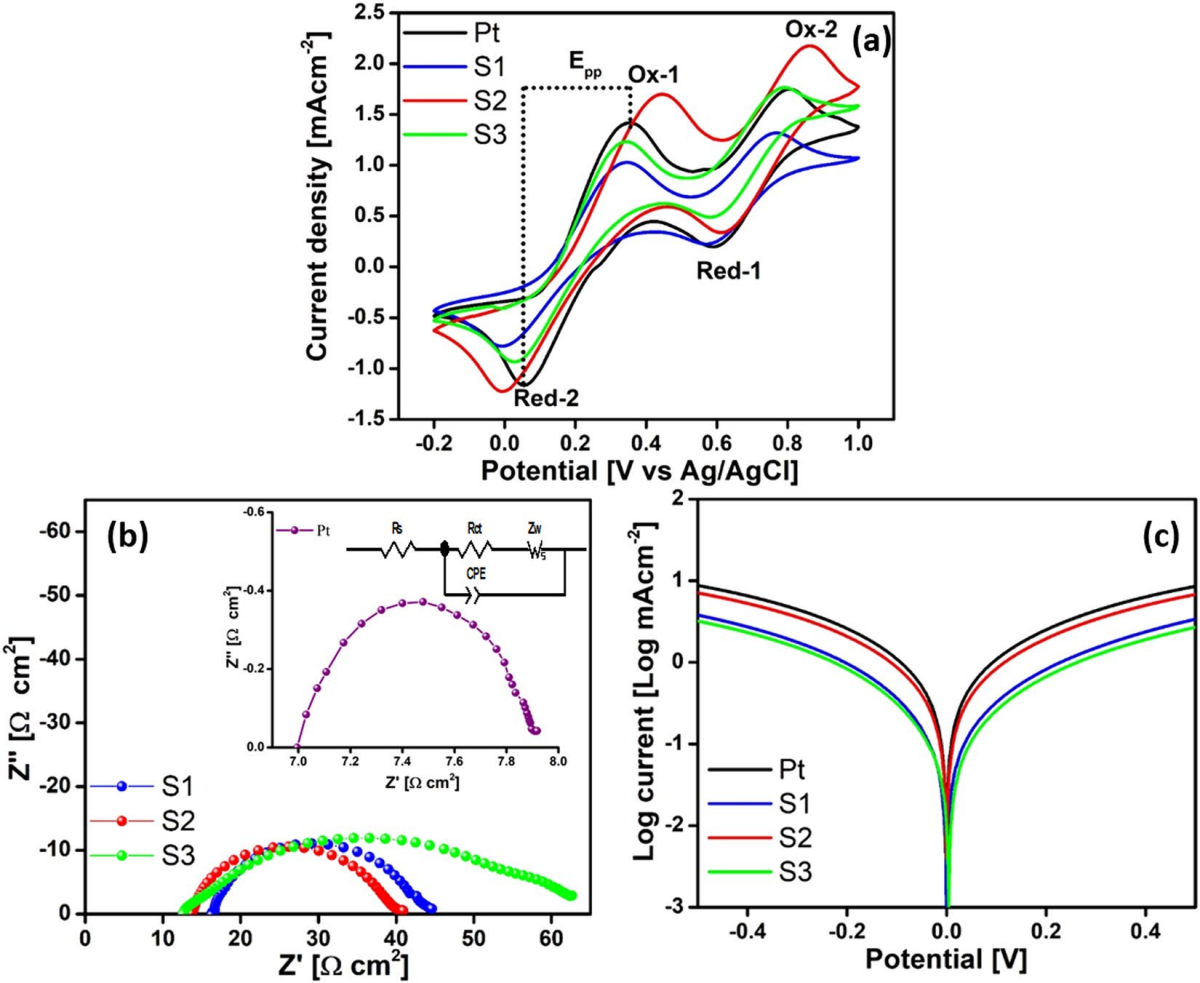
(**a**) CV curves of CEs (scan rate of 20 mVs^−1^). (**b**) Nyquist plots of the symmetrical cells; Inset – equivalent circuit and Nyquist plot of symmetrical cell with Pt (R_ct_: charge transfer resistance, Z_w_: diffusion impedance, R_s_: ohmic internal resistance, CPE: constant phase element). (**c**) Tafel polarization curves of symmetrical cells.

To investigate the application of the MoTe_2_ as a CE in DSSCs, cyclic voltammetry (CV) studies were performed to estimate the reaction kinetics and electrocatalytic performance. CV was conducted using a three-electrode system in an acetonitrile solution consisting of 10 mM LiI, 1 mM I_2_, and 0.1 mM LiClO_4_ at a scan rate of 20 mVs^−1^. Figure 5a shows the CVs of the system for Pt and MoTe_2_ (S1, S2, S3) in the potential interval between −0.2 to 1 V vs. Ag/AgCl. The Ox_1_ and Red_1_ peaks at low potential were attributed to the redox reaction of I_3_^−^ + 2e^−^ ↔ 3I^−^. The Red_1_ peak corresponding to I_3_^−^ + 2e^−^ ↔ 3I^−^ was used to evaluate the integral electrocatalytic ability of CEs to reduce triiodide ions to iodide ions. This reduction occurs in DSSCs, and the current density of this reaction is mainly determined by the number of reduction-active sites on the surface area of the electrocatalyst and the intrinsic electrocatalytic ability of each site. Ox_1_ and Red_1_ represent the same electrochemical reaction I_3_^−^ + 2e^−^ ↔ 3I^−^, in which Ox_1_ indicates the left direction and Red_1_ indicates the right direction.

CV curves show that, like Pt, S1 and S2 also are catalytically active for the reaction that regenerates the redox couple. The higher cathodic peak current density can be used to evaluate the catalytic activity of the CE, and comparable peak current densities imply good electrocatalytic activity. The ~335 nm-thick (S2) CE showed higher current density than the ~185 nm CE (S1), suggesting faster reduction of triiodide ions in the S2 CE compared to the S1 CE (Fig. 4a). The higher cathode current density could be attributed to its relatively higher surface roughness compared to the much smoother S1. Furthermore, S1 and S2 samples displayed similar anodic and cathodic peaks to Pt CE, suggesting that they are active in catalyzing the reduction of I_3_^−^ to I^−^. The peak current and peak to peak separation is important parameters for determining the catalytic activity of CE. The rate constant of a redox reaction is inversely proportional to its peak separation (Epp)^27–29^. Epp is calculated using the formula

1 $${\rm{Epp}}={\rm{Ep}}({\rm{anodic}})\,-\,\mathrm{Ep}({\rm{cathodic}})$$

In DSSCs, the CE has more influence on the negative peak. So, we used this peak for Epp calculations. The Epp for the Pt CE was 295 mV, while those for S1, S2 and S3 were ~354, ~459 and ~308 mV, respectively.

To investigate the electrochemical stability of MoTe_2_ S2 sample and Pt CE, CVs were recorded for 50 consecutive cycles with a potential range from −0.2 to 1 V vs. Ag/AgCl, as presented in Figure S4a. After 50 consecutive scans, the CV shape of sample S2 almost overlapped, and the redox peak current (cathodic and anodic peak current density) for sample S2 was almost constant, which suggests that the MoTe_2_ CE possesses reversible redox activity, good electrochemical and chemical stability, and strong adhesion on the FTO glass substrate. The CVs of sample S1, S2 and S3 were measured using different scan rates from 10 to 100 mVs^−1^ for the (I^−^/I_3_^−^) redox reaction, as shown in Figure S4b–d, respectively. There are a linear increment in the current peak value with increasing scan rate, indicating that the inner sites of MoTe_2_ also become reactive and possess catalyst activity at higher scan rate^29,30^.

To further evaluate the charge transfer kinetics and internal resistance of DSSCs, EIS measurements were performed using symmetric cells fabricated with two identical electrodes (CE/electrolyte/CE). The equivalent circuit model used for fitting the resultant impedance data is illustrated in Fig. 5b. In each curve, there are two well-defined semicircles. The first semicircle at high frequency is related to impedance of charge transfer process occuring at CE/electrolyte and lower frequency range can be assigned to the Nernst diffusion impedance (Z_w_) within electrolyte. The extracted charge–transfer resistance (R_ct_) values of the Pt, S1, S2 and S3 CEs are 0.93, 27.01, 25.97, and 37.44 Ω cm^2^, respectively. The sample S3 has the largest R_ct_ and S2 has the lowest one among the MoTe_2_ samples. R_s_ values of S1, S2, S3 and Pt are 16.47, 13.79, 13.83, and 7.05 Ω cm^2^, respectively. The sample S2 has the lowest R_s_. The R_s_ value of S3 is largest probably due to the largest film thickness.

Tafel polarization analyses were also performed using symmetric cells at a scan rate of 50 mVs^−1^ for Pt, S1, S2 and S3 samples (Fig. 5d). The Tafel curve is usually divided into three regions. The lower potential zone is called the polarization zone, and the middle region (with a sharp slope) is the Tafel zone, which determines the catalytic activity of the electrode. The last zone is the diffusion zone, which determines the diffusion of ions in the electrode. The tangent slope in the anodic or cathodic branch provides information about the exchange current density (J_**0**_) on the electrode^31^. The comparison indicates that S2 (S2 > S1 > S3) is more effective than S1 and S3 at catalyzing the reduction of I_3_^−^. The exchange current density, J_0_ is inversely proportional to R_ct_ from the equation

2 $${J}_{0}=({\mathrm{RT}/\mathrm{nFR}}_{{\rm{ct}}})$$

where R is a gas constant, T is an absolute temperature, n is the number of electrons involved in the reaction, and F is Faraday’s constant^27,32^. A higher J_0_ for Pt and S2 CE implies a lower value of R_ct_ in the impedance measurement.

**Figure 6:**
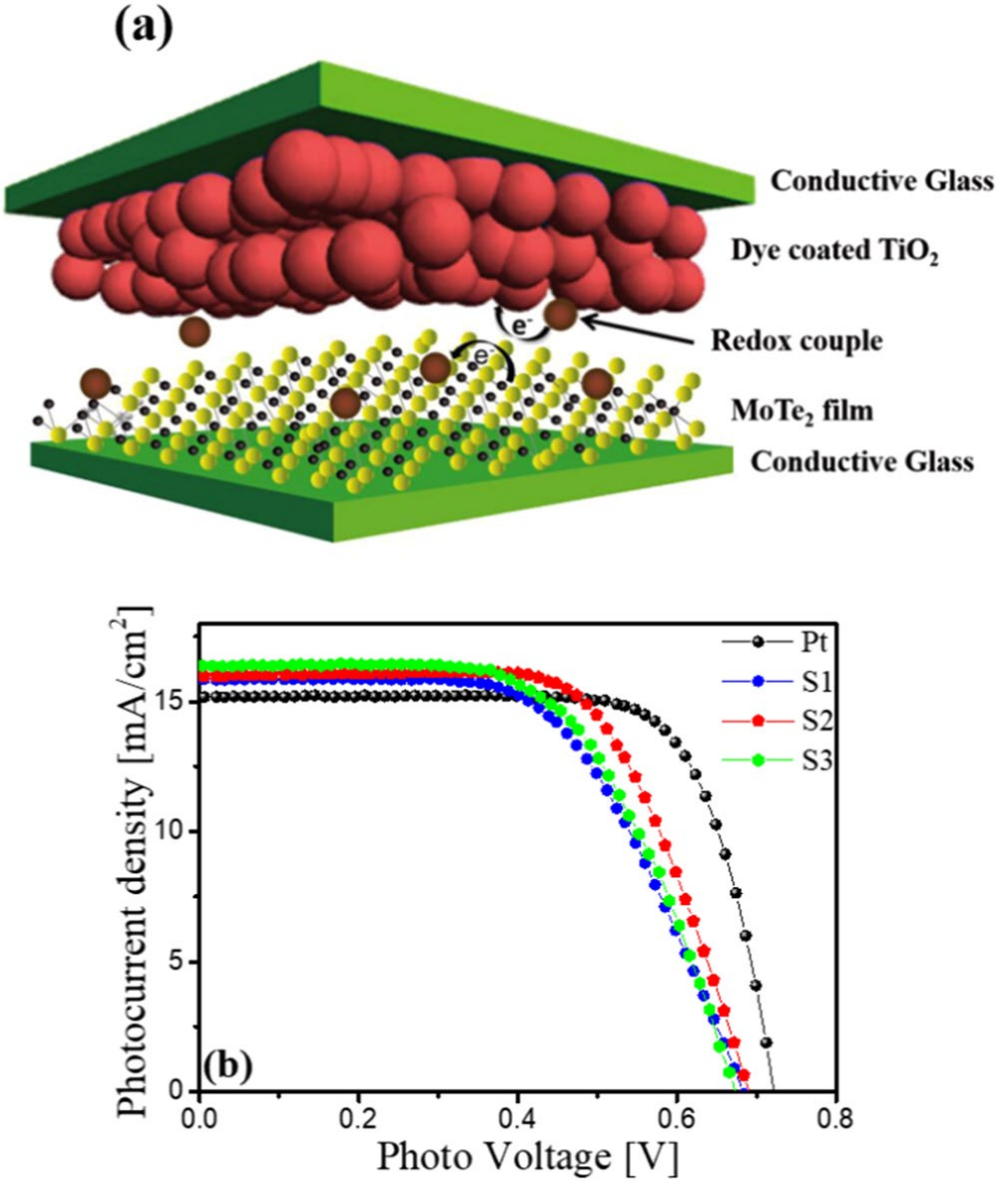
(**a**) Schematic diagram of the electrocatalytic mechanism in DSSC using MoTe_2_ CE. (**b**) Photocurrent–voltage curves of DSSCs with different CEs, measured at AM1.5 G illumination (100 mW cm^−2^).

The schematic of DSSCs with MoTe CE is illustrated in Fig. 6a. The photocurrent density *versus* photovoltage (J-V) curves of the DSSCs are shown in Fig. 6b. The photovoltaic paramters including the short circuit current density (J_sc_), open circuit voltage (V_oc_), fill factor (FF) and PCE (η) of DSSCs with Pt and MoTe_2_ (S1, S2 and S3) CEs under a simulated solar illumination of 100 mWcm^−2^ (AM 1.5) are summarized in the Table 1. The sample S2 CE exhibits the best performnce. The DSSC with S1 CE has lower FF than that with S2 CE, which is related to red-ox behaviour as discussed in earlier. The J_sc_ and FF values are increased for the S2 CE, which leads to enhancing PCE from 6.38% to 7.25%. And, the low efficency of S3 CE is mainly due to low V_oc_ and FF. This could be attributed to higher R_ct_ value as confirmed by EIS analysis.

**Table 1:** Photovoltaic and EIS parameters of Pt, S1, S2 and S3 based DSSC CEs.

Name of CEs	*V*_*oc*_ (V)	*J*_*sc*_ (mA cm^−2^)	*FF* %	PCE (η)%	*R*_*S*_ (Ω cm^2^)	*R*_*ct*_ (Ω cm^2^)	*Z*_*W*_ (Ω cm^2^)
Pt	0.72	15.18	74.37	8.15	7.05	0.93	0.99
S1	0.68	15.84	58.98	6.38	16.47	27.01	27.56
S2	0.69	16.00	65.64	7.25	13.79	25.97	27.51
S3	0.67	16.37	59.50	6.55	13.83	37.44	52.08

The J_sc_ values are decreased in the order of S3 > S2 > S1 > Pt, and PCE values are decreased in the order of Pt > S2 > S3 > S1. It is believed that thick film (S3) could affect electrolyte penetration and result in weaker adhesion to the FTO substrate^33^.

The observed PCE (7.25%) value of the S2 CE was much higher than those of earlier reports based on TMDCs, which include WS_2_ films prepared by a doctor-blading technique (4.56%)^34^, multi-walled carbon nanotubes-MWCNTs@MoS_2_ (6.45%)^35^, multi-wall carbon nanotubes decorated with tungsten sulfide-MWCNTs@WS_2_ (6.41%)^36^, composite films of molybdenum disulfide (MoS_2_)/graphene flakes (5.98%)^29^, and molybdenum disulfide and graphene-MoS_2_/RGO (6.04%)^37^. The variations of V_oc_ and J_sc_ values for MoTe_2_ and Pt CEs can be attributed to the nanoporous nature of the MoTe_2_ CE in contrast to the planar Pt CE, and the high conductivity of Pt. Figures S5 shows incident photon-to current-conversion efficiency (IPCE) curves of DSSCs with the MoTe_2_ CE and Pt CE. These results indicate that catalytic activities depend on the MoTe_2_ thickness since active sites and morphology vary with the growth time, supporting that catalytic activities of thin MoTe_2_ could be modulated by their film thickness and morphology.

## Conclusions

In summary, we presented the sputtering-CVD post annealing route for synthesizing MoTe_2_ as counter electrodes for DSSCs. Detailed electrochemical investigations were carried out using cyclic voltammetry, electrochemical impedance spectroscopy, and Tafel curve analysis to determine the suitability for CE for DSSCs. CV performance revealed that MoTe_2_ CEs possess good electrocatalytic activity and fast reaction kinetics for the reduction of triiodide to iodide. It was found that catalytic activities of thin MoTe_2_ could be modulated by their film thickness and morphology.

The optimum MoTe_2_ CE in a fabricated DSSC exhibited a 7.25% PCE, which is comparable to the 8.15% Pt CE under the same illumination conditions. The presented work suggests that MoTe_2_ would be a promising counter electrode material as a low-cost and highly efficient alternative to Pt in DSSCs.

### Experimental Section and Device preparation

FTO/glass substrates were cleaned with a standard piranha solution and deionized water and were then baked at 120 °C for 5 min. After loading the FTO substrates in a sputter chamber, the chamber was evacuated by a rotary pump and a turbomolecular pump combination to a pressure of ~1 × 10^−7^ torr. Next, Mo thin films were deposited onto FTO/glass substrates using a Mo target (99.99%) by magnetron sputtering. During the film deposition, the Ar gas flow ratio was maintained at 10 sccm, and the power was fixed at 100 W. Mo films were deposited at different sputtering times (such as 20, 30, and 40 min) at room temperature, and these are denoted as S1 (185 nm), S2 (335 nm), and S3 (668 nm) samples, respectively. After removing the samples from the sputter chamber, the as-sputtered films were placed downstream of the chemical vapor deposition (CVD) chamber and heated. The as-sputtered Mo films were annealed in tellurium vapor at 500 °C for 30 min to form MoTe_2_ films and to improve the crystalline quality of the films. A pure tellurium powder (99.99%) was placed upstream of the CVD chamber, and a heating filament for the tellurium boat was fixed at 350 °C. The tellurium powder was evaporated at 350 °C using a mixture of argon and hydrogen (60 sccm - Ar and 30 sccm - H_2_) carrier gases, and the pressure of the CVD chamber was kept at 2 × 10^−2^ Torr.

### Fabrication of DSSCs

DSSCs were fabricated to evaluate the CE performance of the MoTe_2_ films using our method^38–41^. Briefly, thin blocking layer TiO_2_ was deposited onto a cleaned FTO glass substrate (15 × 15 mm^2^) by dipping it in 40 mM TiCl_4_ solution for 30 min at 70 °C and annealing it at 450 °C for 30 min. A homemade titanium dioxide (TiO_2_) powder paste of P25 was coated on the cleaned FTO glass as the main layer (~12 µm thickness) using a simple doctor blade coating technique. The TiO_2_-coated FTO was then sintered in five steps of 70, 325, 375, 450, and 500 °C for 30, 5, 5, 15, and 15 min, respectively, in a high temperature furnace (Lab House Co.). Additionally, a scattering layer (~6 µm) was coated over the main layer and sintered using the same sintering steps. The TiO_2_ film was then sensitized with 0.5 mM N 719 prepared in an absolute ethanol: acetonitrile (1:1) solution for 24 h. The polymer electrolyte, which was composed of 0.5 M LiI, 0.6 M 1-propyl-2,_3_^−^dimethylimidazolium iodide, 0.05 M I_2_, 0.5 M 4-tert-buylpyridine, and 3% w/w polyethylene oxide (Mw 250,000) with acetonitrile as the solvent was then injected between the two electrodes. The Pt-coated CE was prepared by spreading a drop of 2 mM chloroplatinic acid hexahydrate (H_2_PtCl_6_) in isopropanol onto the FTO substrates using a simple brush method and heating it to 400 °C for 15 min in ambient air^42,43^. The dye-sensitized TiO_2_ photoanode with an active area of 0.25 cm^2^ and the as-fabricated CE were assembled using a 50-μm-thick spacer made of polyimide adhesive tape.

## Electronic supplementary material

supporting information
