# Supplementary material for: Large area growth of MoTe2 films as high performance counter electrodes for dye-sensitized solar cells
Source: Sci Rep. 2018 Jan 8;8:29. doi: 10.1038/s41598-017-18067-6 (PMC5758823; doi:10.1038/s41598-017-18067-6)
Supplement: Supplementary file 1 — supporting information [file 41598_2017_18067_MOESM1_ESM.doc]

**Electronic Supplementary Information(ESI)**

**Large area growth of MoTe_2_ films as high performance counter electrodes for dye-sensitized solar cells**

Sajjad Hussain^a, b†^, Supriya A. Patil^c, d†^, Dhanasekaran Vikraman^e^, Naveed Mengal^f^, Hailiang Liu^a, b^, Wooseok Song^g^, Ki-Seok An^g^, Sung Hoon Jeong^f^, Hak-Sung Kim^c, d^*, and Jongwan Jung*^a,b^

^a^Graphene Research Institute, Sejong University, Seoul 143-747, Republic of Korea

^b^Institute of Nano and Advanced Materials Engineering, Sejong University, Seoul 143-747, Republic of Korea

^c^Department of Mechanical Engineering, Hanyang University, Haengdang-dong, Seongdong-gu, 133-791 Seoul, Republic of Korea

^d^Institute of Nano Science and Technology, Hanyang University, Seoul 133-79, Republic of Korea

^e^Division of Electronics and Electrical Engineering, Dongguk University-Seoul, Seoul 04620, Republic of Korea

^f^Department of Organic and Nano Engineering, Hanyang University, Seoul 133-791, Republic of Korea

^g^Thin Film Materials Research Group, Korea Research Institute of Chemical Technology, Daejon 305-600, Korea

^†^These authors are equally contributed to this work.

*Corresponding authors E-mail:

[jwjung@sejong.ac.kr](mailto:jwjung@sejong.ac.kr) (Prof. Jongwan Jung), [kima@hanyang.ac.kr](mailto:kima@hanyang.ac.kr) (Prof. Hak-Sung Kim)

KEYWORDS: MoTe_2_, Magnetron sputtering, CEs, DSSC.

**~~
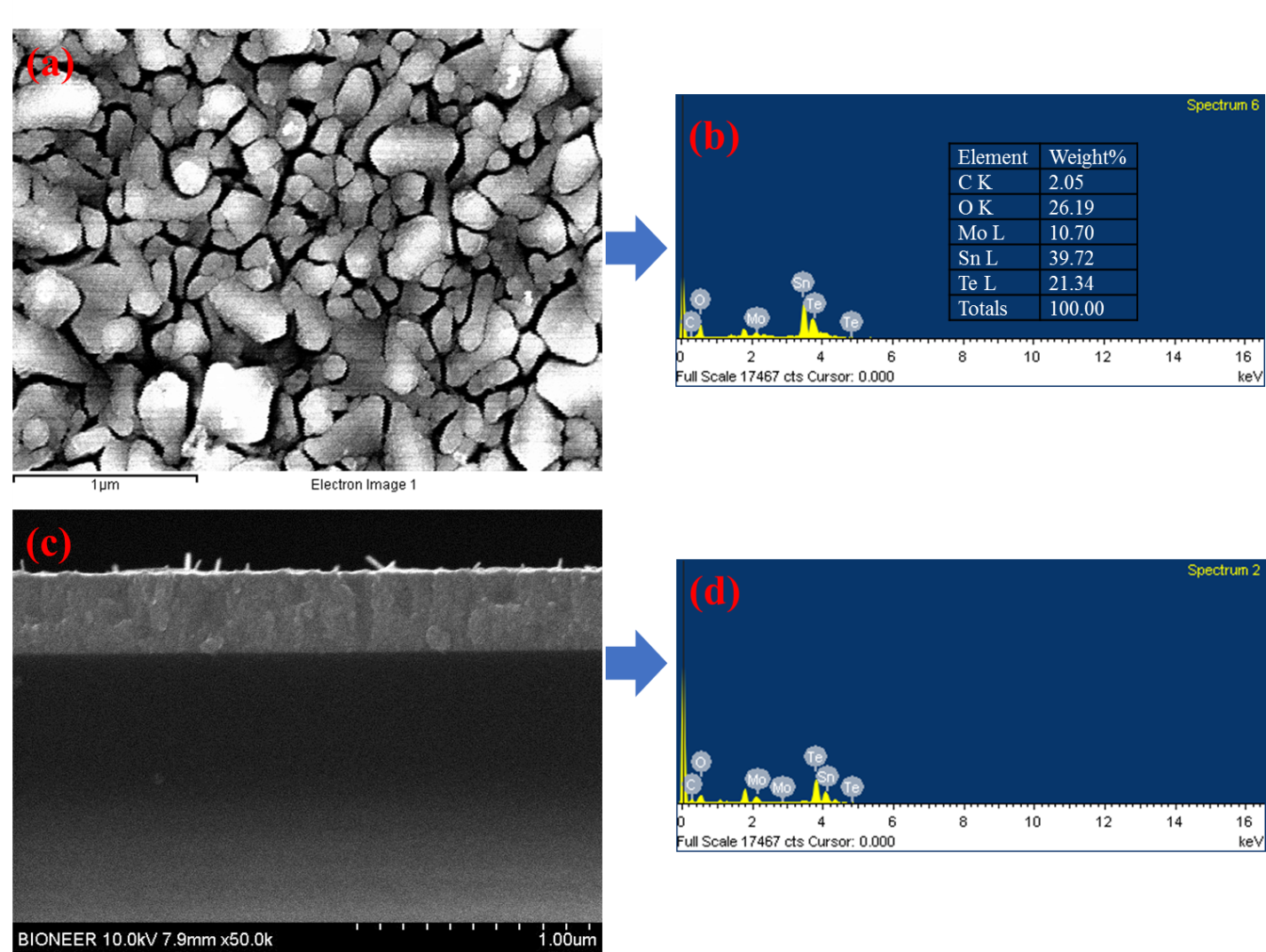
~~**

Figure S1. (a) Top surface FE-SEM image and (b) corresponding EDX pattern, (c) cross sectional FE-SEM image and (d) corresponding EDX pattern for the MoTe_2_ sample S2.


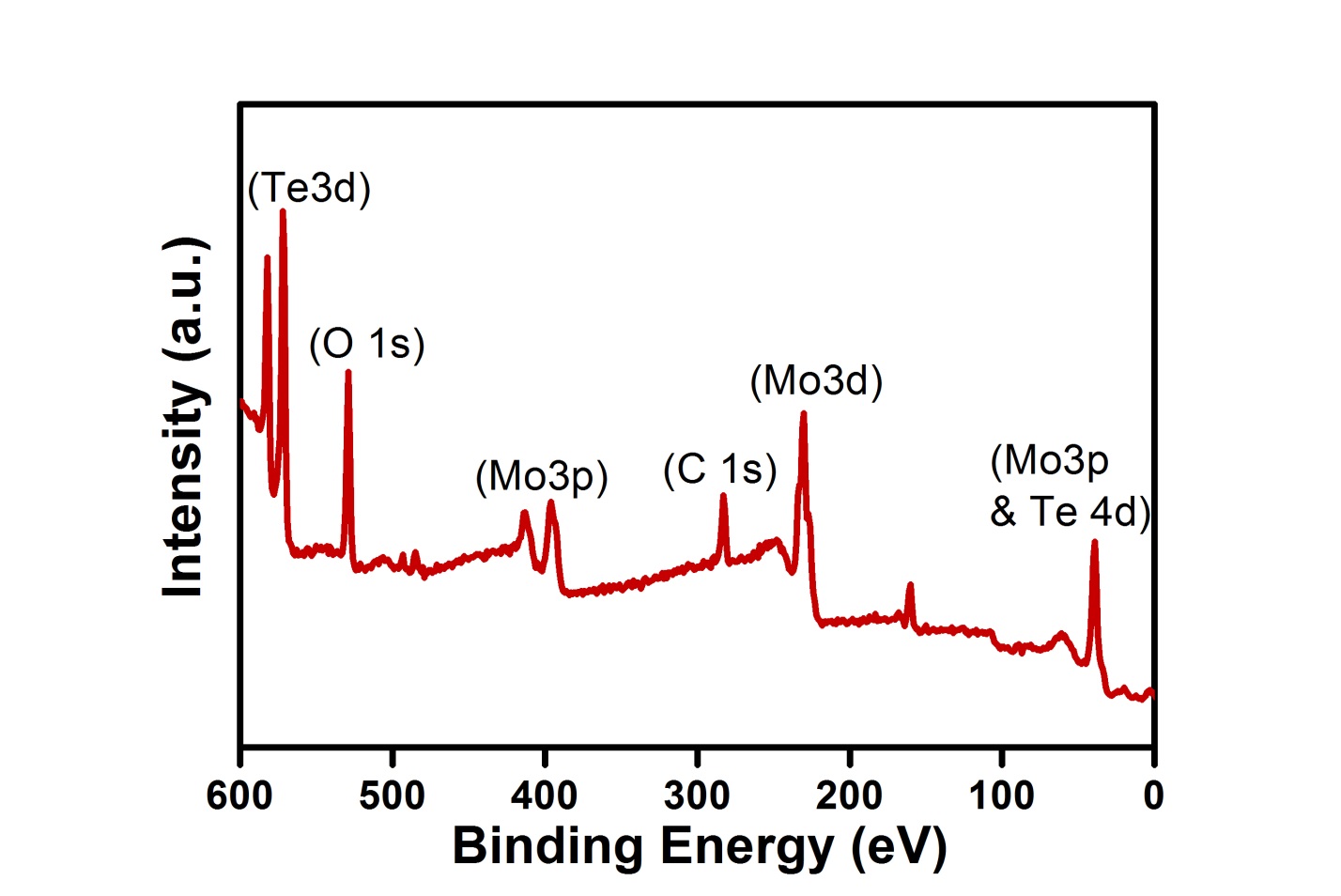


**Figure S2**. XPS survey spectrum of MoTe_2_ sample S2.


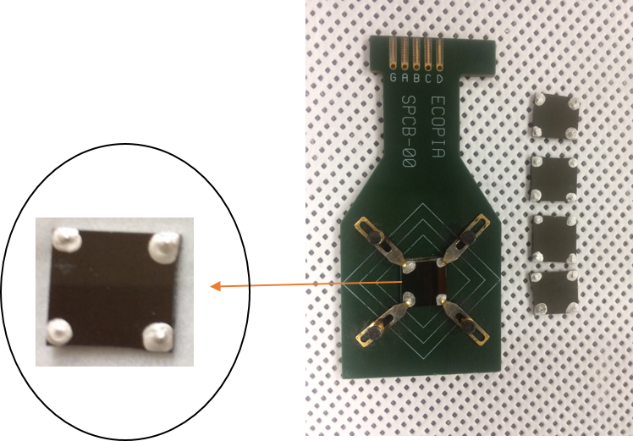


| **Name** | **Conductivity (Ω-cm)^-1^** | **Surface hole concentration**  **/cm^2^** | **Mobility**  **cm^2^/Vs** | **Type** | **Literature reference** |
| --- | --- | --- | --- | --- | --- |
| **MoTe_2_**  **_(S2)_** | 3.3x10^-1^ | 3.2x10^11^ | 95 | P-Type | P-Type |

**Figure S3**. Hall effect measurement for S2 MoTe_2_ CE.

**
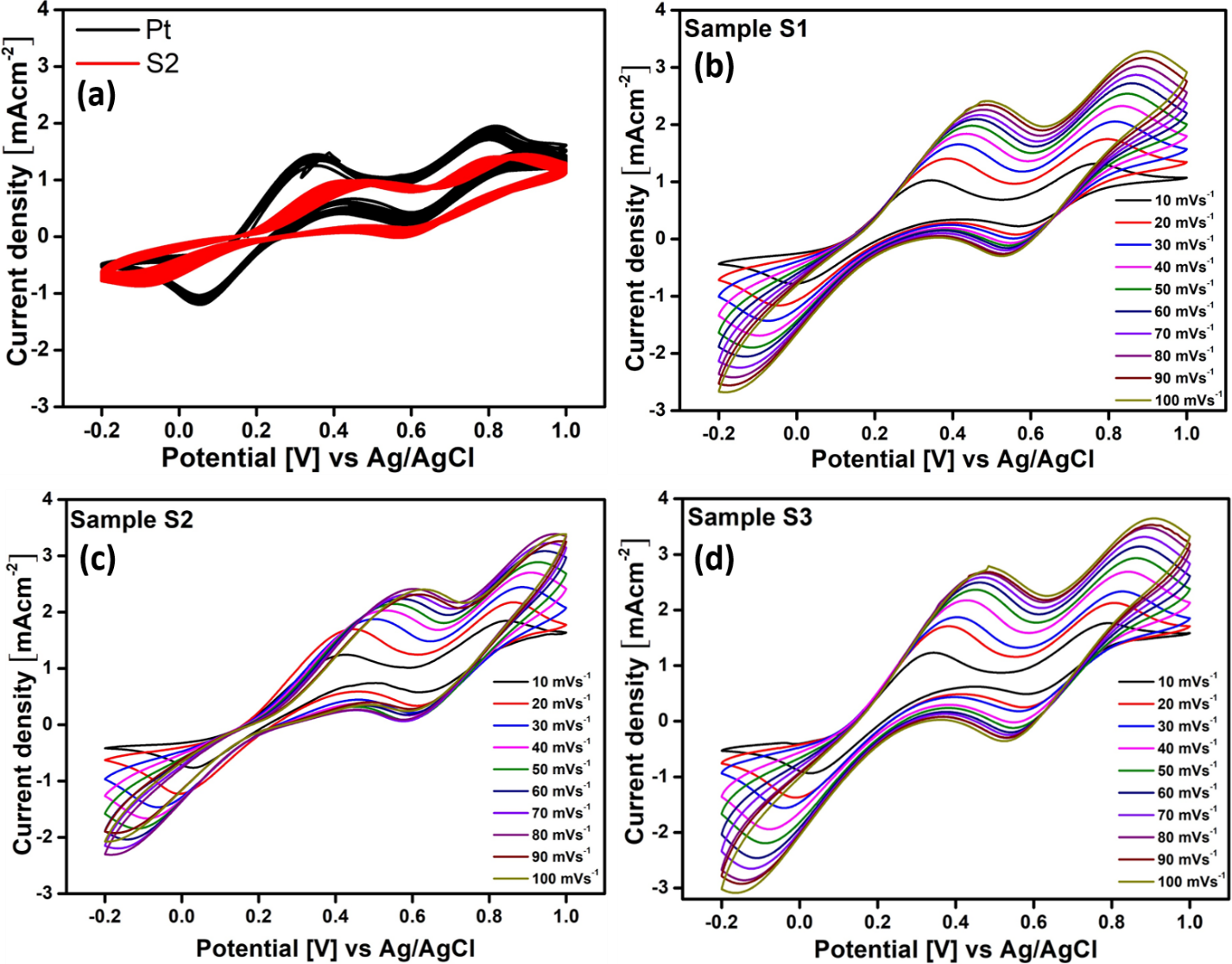
**

**Figure S4**. (a) CV of stability analysis for Pt and MoTe_2_ S2 samples for 50 consecutive cycles at a 20 mV s^-1^ scan rate; (b-d) CV at various scan rates for (b) sample S1 (c) sample S2 and (d) sample S3.


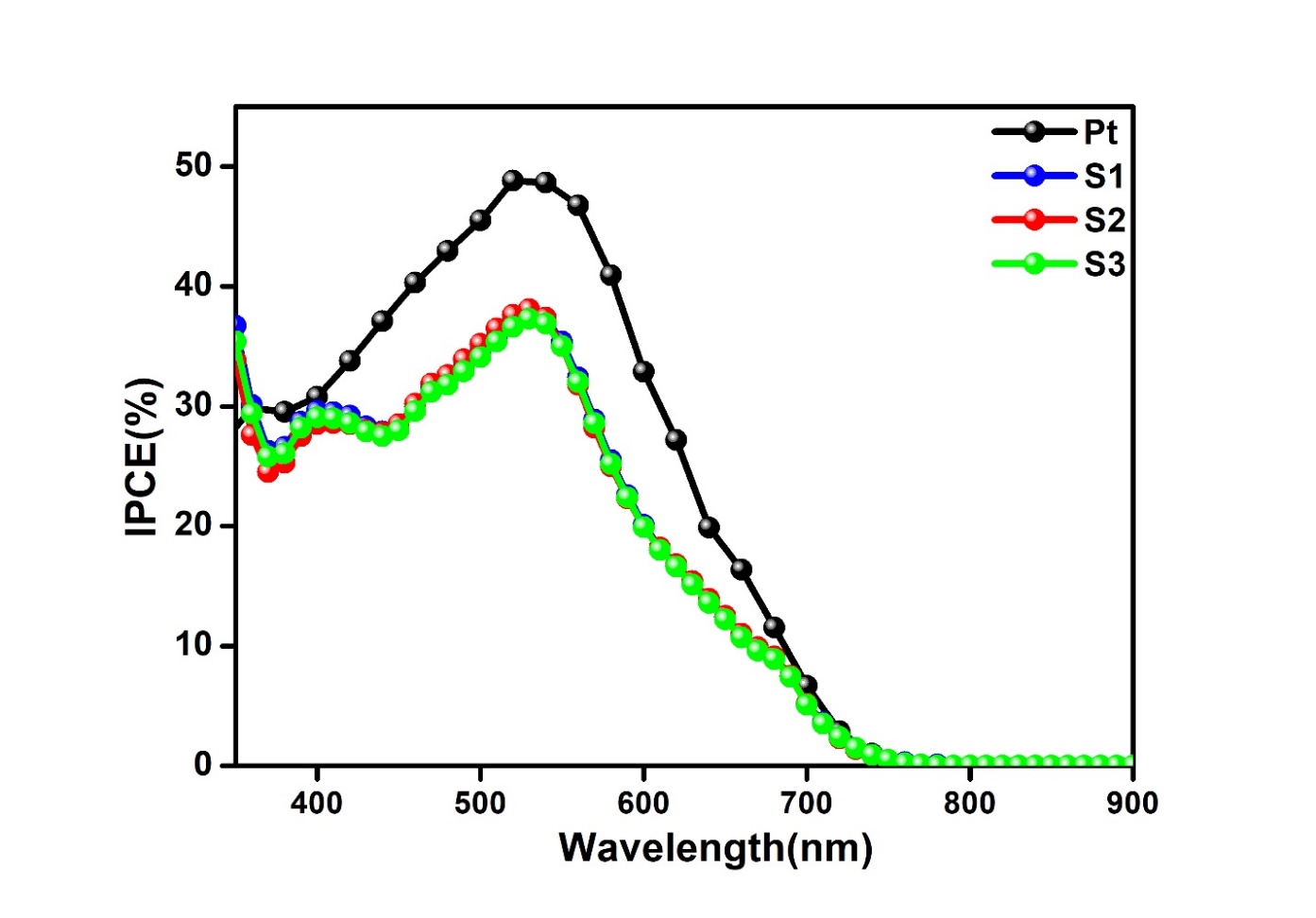


**Figure S5.** IPCE spectra of TiO_2_ DSSCs employing MoTe_2_ CE and Pt CE.
